# The materials tetrahedron has a “digital twin”

**DOI:** 10.1557/s43577-021-00214-0

**Published:** 2022-02-01

**Authors:** Michael E. Deagen, L. Catherine Brinson, Richard A. Vaia, Linda S. Schadler

**Affiliations:** 1 Department of Mechanical Engineering, The University of Vermont, Burlington, VT 05405 USA; 2 Department of Mechanical Engineering and Materials Science, Duke University, Durham, NC 27708 USA; 3 Air Force Research Laboratory, Wright-Patterson Air Force Base, OH 45433 USA

**Keywords:** Informatics, Data/database, Life cycle, Computation/computing, Artificial intelligence, Education

## Abstract

**Abstract:**

For over three decades, the materials tetrahedron has captured the essence of materials science and engineering with its interdependent elements of processing, structure, properties, and performance. As modern computational and statistical techniques usher in a new paradigm of data-intensive scientific research and discovery, the rate at which the field of materials science and engineering capitalizes on these advances hinges on collaboration between numerous stakeholders. Here, we provide a contemporary extension to the classic materials tetrahedron with a dual framework—adapted from the concept of a “digital twin”—which offers a nexus joining materials science and information science. We believe this high-level framework, the materials–information twin tetrahedra (MITT), will provide stakeholders with a platform to contextualize, translate, and direct efforts in the pursuit of propelling materials science and technology forward.

**Impact statement:**

This article provides a contemporary reimagination of the classic materials tetrahedron by augmenting it with parallel notions from information science. Since the materials tetrahedron (processing, structure, properties, performance) made its first debut, advances in computational and informational tools have transformed the landscape and outlook of materials research and development. Drawing inspiration from the notion of a digital twin, the materials–information twin tetrahedra (MITT) framework captures a holistic perspective of materials science and engineering in the presence of modern digital tools and infrastructures. This high-level framework incorporates sustainability and FAIR data principles (Findable, Accessible, Interoperable, Reusable)—factors that recognize how systems impact and interact with other systems—in addition to the data and information flows that play a pivotal role in knowledge generation. The goal of the MITT framework is to give stakeholders from academia, industry, and government a communication tool for focusing efforts around the design, development, and deployment of materials in the years ahead.

**Graphic abstract:**

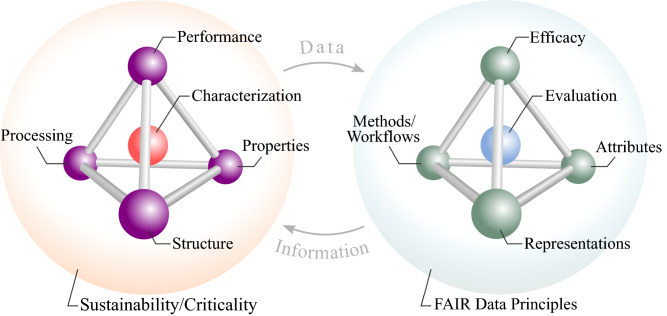

## Introduction and background

**Figure 1:**
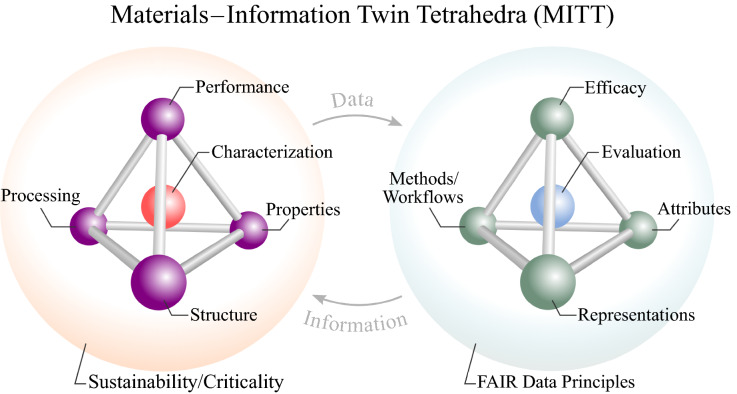
Materials–information twin tetrahedra (MITT) framework translates foundational concepts in materials science and engineering (from the materials tetrahedron) to parallel notions in information science (the “information tetrahedron”), highlighting the data and information flows that form a closed-loop for knowledge creation around the discovery, design, development, and deployment of materials.

Human technological evolution and innovations in materials share an interwoven history punctuated by advances in tools, instrumentation, and the exchange of knowledge. Today, as digitization and automation drive down the marginal cost of collecting, archiving, and sharing data, methods for extracting value from this abundance of digital information have proliferated. With a new paradigm of data-intensive scientific research and discovery underway,^1^ the field of materials science and engineering aims to drastically reduce the overall time and cost to discover and develop new materials through efforts such as the Materials Genome Initiative.^2^ While an ecosystem of data repositories and e-collaboration platforms offers numerous tools and resources around materials data,^3–7^ facilitating interoperability and integration into the typical materials research workflow remains an ongoing challenge. To broaden the cross-fertilization of solutions across materials sub-fields and information technologies, a shared conceptual framework would enable experts in various sub-disciplines to leverage, and contribute to, their combined abilities in order to solve challenges in an integrated, interoperable, open architecture. Fundamentally, we postulate that such a foundational framework lies within the complex interplay between materials and information science. We propose that an extension of the classic materials tetrahedron framework—inspired by the notion of a “digital twin”—provides such a scaffold as the materials–information twin tetrahedra (MITT) (**Figure ****1**). Here, we deliver a perspective on the convergence of materials and information science through the lens of this new MITT framework, and in this perspective article we highlight select works and recent reviews as illustrative examples in this emerging and evolving space.

Since its inception, the materials tetrahedron has remained an enduring visual icon that illustrates the interdependence of key concepts in materials science and engineering. Published in 1989 by the National Research Council in its report on materials science and engineering for the 1990s,^8^ the symbolic polyhedron depicts four foundational elements of materials science and engineering while emphasizing the edges that connect them. These four elements—processing, structure, properties, and performance—have also been arranged linearly as a three-link chain to highlight the forward “cause and effect” progression from processing to performance as well as its inverse “goal/means” counterpart.^9,10^ In 2008, a depiction of the materials tetrahedron with characterization as an interstitial element at its center was added to the public domain by means of Wikipedia.^11^ In 2019, Donahue made the case for appending the dimension of sustainability/criticality to the materials tetrahedron for a holistic perspective on the roles that materials play in the anthroposphere.^12^ Donahue’s reimagination of the materials tetrahedron paradigm extends beyond individual use cases of materials by recognizing factors such as raw material supply risk, price volatility, and the environmental implications of materials in their full life cycle.^1^ At its essence, sustainability/criticality addresses the extent to which our materials systems, throughout their life cycle, impact and interact with other systems.

**Figure 2:**
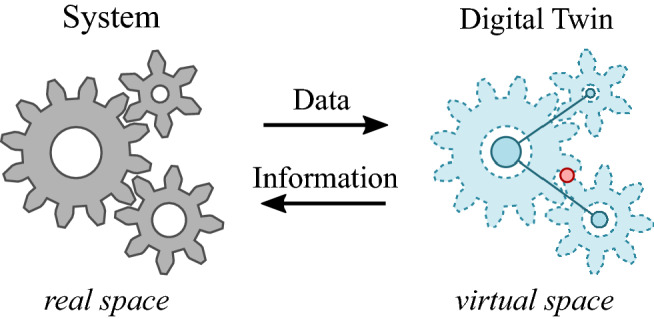
A digital twin comprises a virtual representation of a real system, linked by continual data and information flows throughout the system’s life cycle.

Over the past two decades, a concept has emerged from the manufacturing community that elegantly bridges the gap between physical systems and our virtual representations of them. Originally described by Grieves in the early 2000s as product life cycle management (PLM),^13^ the term “digital twin” was coined by Vickers et al. in 2010 in a NASA technology area roadmap for materials, structures, mechanical systems and manufacturing.^14^ The digital twin—a virtual representation of a system that exists alongside its physical counterpart throughout its life cycle—includes (1) the sensor-equipped physical object or system itself; (2) a high-fidelity virtual representation, including historical data and predicted future performance; and (3) the data and information flows between the physical world and the digital twin, often through multi-modal sensing and probabilistic modeling at the instance and aggregate level (**Figure ****2**).^15,16^ The value proposition of a digital twin stems from its ability to manage and apply heterogeneous information, and the concept has gained significant traction in recent years as evidenced by citations in the literature.^17,18^ Manifestations of digital twins have appeared in manufacturing,^19–21^ aerospace,^15,22^ healthcare,^23–25^ transportation,^26–29^ and the built environment including utilities and smart cities.^30–35^ The concept will continue to gain traction as stakeholders adopt common data standards and models, improve data sharing practices, develop products and services around digital twins, and establish forums for experts in various disciplines (including materials science) to meet and collaborate.^36^

When we refer to a “digital twin” for the materials tetrahedron—a purely conceptual framework, as opposed to a physical system— we describe its interdependent counterpart in information science, hereby referred to as the “information tetrahedron.” Given the data and information flows that connect these conceptual frameworks and provide the basis for knowledge creation, the digital twin analogy remains apropos. For reference, the terms data, information, and knowledge represent levels in the *data-information-knowledge-wisdom* (DIKW) hierarchy.^37^

In this perspective, we offer the MITT framework as a means to contextualize the interdisciplinary efforts across various stakeholders in furtherance of a robust infrastructure and workforce around materials data. In the following sections, we articulate the meta-framework underpinning MITT, describe the components of the information tetrahedron, and illustrate one possible application of the dual framework with existing and upcoming technologies. We believe that students, practitioners, educators, and policymakers armed with this framework can apply modern and future digital information capabilities to address grand materials challenges with the high-level perspective that has guided and benefited the field of materials science and engineering for more than three decades.

## Translating the materials tetrahedron to information science

In a prototypical implementation of the MITT framework, materials systems provide data to information systems, which in turn generate information that guide the further improvement of these materials systems. Iteratively, this cycle aims for knowledge creation around discovery, design, development, and deployment of materials systems. Humans necessarily remain in the loop in terms of strategic guidance and implementation of this process, but the reciprocal exchange of data and information should take advantage of available automated workflows.

**Figure 3:**
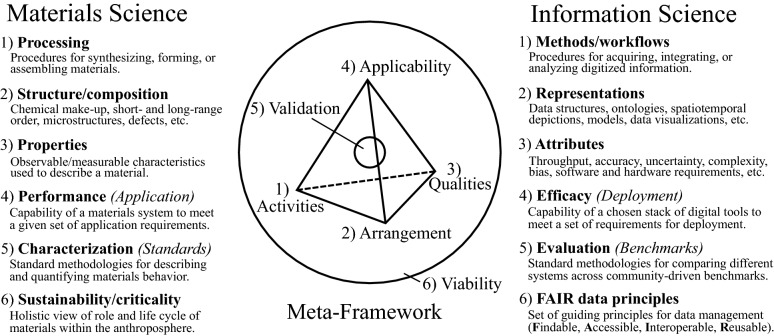
An underlying meta-framework captures the elements of the (extended) materials tetrahedron and relates these elements to counterparts in information science.

To define the information tetrahedron, we identified six dimensions underpinning the materials tetrahedron and translated these dimensions to notions from information science (**Figure ****3**). These dimensions—activities, arrangement, qualities, applicability, validation, and viability—form a generalizable meta-framework at the heart of the proposed MITT framework.

The meta-framework classifies the various aspects of these frameworks broadly, such that the specification and implementation of instantiations of this meta-framework may follow the best practices of their respective fields. Thus, the meta-framework enables both the materials tetrahedron and information tetrahedron to describe individual aspects of each domain without relying on one-to-one mappings. For example, the dimension “Arrangement” refers to materials structure and composition in the materials domain and to digital representations including data and metadata structures in the information domain. The generalizability of this meta-framework may enable the tetrahedron concept to percolate into other domains, providing a useful organizational and translational tool. In the following section, we expound further on the components of the information tetrahedron of the MITT framework, focusing on recent progress in materials data and informatics.

## Components of the information tetrahedron

Strictly speaking, a digital twin represents a virtual instantiation of a physical system. To translate the essence of a “digital twin” to the materials tetrahedron—a purely conceptual framework to begin with—we describe the MITT framework as a paradigm that considers both materials science and information science side-by-side, connected by data and information flows. Development of materials data and informatics systems involves many complex tradeoffs and nuances that accompany the various strategic and design decisions made by software architects and engineers.^38^ Striving for a general-purpose digital information system while managing scope to stay within development constraints mirrors many of the challenges faced by materials engineers in designing or optimizing materials systems. **Table** **I** summarizes the following paragraphs, which relate the information science components of the MITT paradigm to recent progress in materials data and informatics.

**Table I:** Contextualized examples, including recent progress in materials data and informatics, related to each element of the information tetrahedron of the MITT framework.

*Activities*	*Arrangement*	*Qualities*	*Applicability*	*Validation*	*Viability*
Methods/ Workflows	Representations	Attributes	Efficacy	Evaluation	FAIR Data Principles^39^
Inverse design^40^ Multiphysics simulations^41^ Autonomous experiments^42–44^ Interpretable ML methods^45,46^ Open-source toolkits^47–49^ Correlative characterization^50^ Mixed-initiative user interaction^51^	Atomic or molecular data structures^52,53^ Spatiotemporal depictions (pixelated, voxelated, graph-based) Physical descriptors^54^ Schemas, taxonomies, controlled vocabularies^55,56^ Workflow representations^57^ Ontologies^58^ Low-dimensional embeddings Data visualizations	Complexity Throughput Accuracy Bias Uncertainty Usability Software dependencies Hardware requirements Cost	Clearly defined scope and requirements Extent to which system meets requirements Suitability of system for the task at hand Time and cost savings over alternatives	Benchmark data sets and tasks^59,60^ Objective tests and measures for comparison Metrics for data “FAIR-ness” UI/UX assessment Validation of predictions	Findable Accessible Interoperable Reusable AI-ready^61^ Sustained life cycle efficacy

### Methods/workflows

Acceleration of materials discovery and development presupposes the accompanying digital methods and workflows for collecting, curating, integrating, and analyzing data. Methods and workflows may include algorithms for achieving “inverse design” of processing parameters given a set of performance goals,^40^ multiphysics simulations,^41^ high-throughput methods that automate or semi-automate experimentation and data collection,^42–44^ interpretable machine learning methods,^45,46^ retrieval and management of large data sets facilitated by open-source toolkits,^47–49^ methods for bridging length scales and imaging modalities via correlative characterization,^50^ or mixed-initiative user interfaces that leverage automation to support better decision-making through human–computer interaction.^51^ When applying the MITT paradigm to a materials data and informatics, one should consider how these methods and workflows combine to transform fragmented data into actionable information.

### Representations

As symbolic representations of information, data derive their utility only when placed within broader models or contexts that impart meaning. In the absence of a preexisting model of the world, machines do not have the innate capacity to interpret data without some form of metadata (“data describing the data”), necessitating efforts from the community to develop and adopt common standards around digital representations for materials data and metadata. These representations may include: data structures for atomic or molecular arrangements;^52,53^ pixelated, voxelated, or graph-based spatiotemporal depictions at various hierarchical scales; quantitative physical descriptors;^54^ schemas, taxonomies, and controlled vocabularies;^55,56^ or standardized representations of materials processing or computational workflows.^57^ Metadata structures include ontologies,^58^ which comprise machine-interpretable networks of linked concepts, as well as structured representations of data provenance. Data that are inherently uninterpretable (e.g., a trained neural network model) may have metadata describing their inputs and outputs or low-dimensional embeddings serving as representations. To display collected or analyzed data, advances in interactive data visualization can provide an interface between these systems and human decision-makers. When applying the MITT framework, one should carefully consider (meta)data representations as they relate to deployment requirements, with a preference for representations aligned with the FAIR data principles (findable, accessible, interoperable, reusable).^39^

### Attributes

The attributes of a digital information system should provide an objective view of the system and its standing among comparable systems. These attributes include technical specifications of the software and hardware, as well as software libraries or data corpora that these systems rely on. Complexity, throughput, accuracy, bias, uncertainty, usability, and other relevant system attributes should be accompanied by community-driven benchmarks that enable researchers to objectively measure them so that system architects and end users can select the best stack of digital tools given their budget and scope. In the context of MITT, attributes reflect the various tradeoffs of particular methods, workflows, and representations at the individual and systems level.

### Efficacy

The efficacy of an information system rests on its architecture, integration, and environment in the context of a given deployment setting. In addition to the system’s internal ability to process and analyze data, efficacy dictates how well the system can interface with the real world by incorporating new data from external signals and presenting actionable information to decision-makers. Tradeoffs between system components must be considered when optimizing system efficacy.

### Evaluation

Community-driven standards and procedures for objective validation of digital information systems lie at the heart of the information tetrahedron. Benchmark data sets and tasks facilitate consistent and systematic evaluation of the myriad digital information methods and systems that will emerge in the coming years.^59,60^ Evaluation protocols and benchmarks for FAIR data management as well as human usability should be considered among these assessments.

### FAIR data principles

Conversations around the life cycle and impact of data and information remain pivotal in order to ensure continual growth and reuse of information resources. The four guiding principles for data management and stewardship that comprise FAIR (findable, accessible, interoperable, reusable) recognize the need among stakeholders in academia, industry, funding agencies, and publishers for an infrastructure that maintains the value of data beyond initial publication.^39^ Ongoing, community-wide commitments to FAIR data and metadata standards aim to reduce the labor-intensive data pre-processing and cleaning steps that historically kick off data science projects, making data “AI-ready.”^61^ Some data may remain proprietary or confidential, but any data advertised as “open” should contain adequate metadata. In addition to mandates or incentive structures,^62^ the prevalence of FAIR data depends on automated methods and workflows that enable researchers to seamlessly manage and share their data. As a supplement to the FAIR principles for data, materials data and informatics platforms should incorporate configuration management activities to maintain continued efficacy and integrity throughout the projected life cycle of the platform. When considering an information system’s viability, one must also take into account internal factors such as reliance on specific software and hardware infrastructures in addition to external factors such as funding agency mandates and new expectations driven by transformations in data culture.

## Highlighting recent progress and reviews

**Figure 4:**
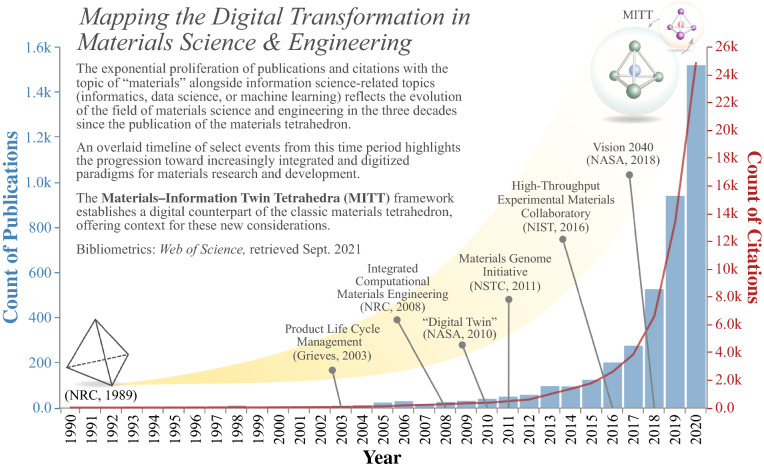
Bibliometric data from *Web of Science* show the count of publications (blue bars) and citations (red line) at the intersection of the topic of “materials” with any of the topics of “informatics,” “data science,” or “machine learning” in the years 1990–2020. The timeline highlights select examples from this progression toward increased integration and digitization in materials research and development.

The topics of informatics, data science, and machine learning have appeared with increasing prevalence in materials-related works in the academic literature—most notably in the past decade—as reflected in the quantity of publications and citations (**Figure ****4**). This exponential growth persists even when normalized by the steady annual growth of all materials-related publications. The progression shows the extent to which the field of materials science has evolved in the three decades since the initial conception of the materials tetrahedron in 1989 and the impact of integration and digitization on the landscape of materials research and development. For example, the availability and capability of computational tools initiated the discipline of integrated computational materials engineering (ICME),^63^ combinatorial materials synthesis and rapid characterization techniques led to high-throughput experimental (HTE) methodologies,^64^ and model-based concurrent design, development, and deployment for materials and systems form the basis of NASA’s recent “Vision 2040” roadmap.^65^

Systems for autonomous experimentation (AE) exemplify the closed-loop coupling of materials science and information science as laid out in a recent community perspective article by Stach et al.^66^ Owing to advances in mechanical automation and robotics, rote tasks that often consume researcher time and effort can be delegated to machines that prepare, characterize, and test samples. Aided by machine learning, collected data inform future hypothesis testing for efficient navigation of complex, high-dimensional design spaces. Researchers may instead focus on higher-level guidance such as providing insights, context, and objectives to the AE campaign. Stach et al. describe the benefits of AE in terms of multiplying the productivity of individual researchers in addition to network effects when these distributed systems share learned insights. Importantly, they present areas for investment in hardware, software, data management, and workforce education in the coming years to realize the potential of AE systems for materials research and development. Similar opportunities and challenges for closed-loop automation exist outside the field of materials science as well, such as drug discovery,^67^ healthcare,^68^ supply chain management,^69^ architecture,^70^ and chemistry.^71,72^

Although an exhaustive review falls outside the scope of this perspective, we urge the interested reader to consult recent review articles and perspectives that explore the intersection of materials science with one or more of the topics in information science outlined in this perspective. These resources include discussions of data ecosystems and infrastructures for metadata management;^73–75^ high-throughput library generation and characterization;^76,77^ successes and challenges of Materials Genome Initiative research, machine learning methods, and computational materials databases;^78–80^ methods for linking materials characterization and computation across length scales;^81,82^ methods, representations, and applications in the field of polymer informatics;^83^ materials discovery for energy applications;^84^ and integrated systems for next-generation microscopy.^85^

## Demonstrating the value of materials data and informatics

Just as the classic materials tetrahedron does not fully capture, for example, the subtleties of precipitation hardening of aluminum alloys, the MITT framework does not claim to solve the challenges ahead in creating a robust infrastructure around materials data and informatics. Instead, the framework presents a holistic view of certain high-level, interdependent elements such that experts across disciplines can make this infrastructure a reality. The broader materials community will accept and adopt materials data and informatics resources as these resources demonstrate more efficient digital representations and methods, offer robust cyber-physical infrastructures that establish trust, and provide training of the workforce in the utilization of these tools.^86^ Making these systems and tools relevant to the broader materials community will require clear articulation of the value proposition of these resources, continued investment in the areas laid out in the MITT framework, and the refinement of shared visions for the future of materials research and development.

Data and informatics have the potential to transform the conventional landscape of materials research and development. By extrapolating from existing technologies, one can speculate on manifestations of the materials research landscape in the near future:

*…A researcher monitors the incoming data on her tablet. In the background, an autonomous research system chirps and hums as it passes cassettes of samples between preparation stages for characterization. She, along with a geographically distributed network of colleagues, designs and develops materials systems for electrically insulating coatings—her materials system of choice features functional molecules tethered to nanoscopic ceramic particles and dispersed in self-healing polymers. The boundless configurations of possible materials components make exploration of the design space both invigorating and daunting. To manage the complex design tradeoffs, she and her team develop methods that consolidate computational and experimental research—augmented with troves of data from several online data repositories through APIs—to predict the electrical, mechanical, thermal, and degradation behavior of these materials systems. To validate and improve model predictions along the Pareto frontier, she runs combinatorial experiments with gradient libraries that vary mix ratios and process parameters to reveal and quantify the effects on nanoparticle dispersion in these composite systems. On some days, carrying out an experiment involves a few simple gestures on her tablet—unified data formats enable the autonomous research system to interoperate with the workflow planning system, lab inventory, characterization and image analysis tools, and her team’s Bayesian models—and each material sample receives a globally unique identifier with semantic links to its detailed processing history and characterization results. As the data populate her customized dashboard of interactive charts in real-time, she recalls stories of data antiquity—misplaced USB drives, critical experimental parameters scrawled in the margins of laboratory notebooks, and hours spent manually aggregating data from PDF files into spreadsheets. When she considers how she cites and publishes work today, everything just feels a lot more—what’s the right word?* Natural, *she mutters to herself. With a few touches on her tablet, she pushes the newly acquired data to her team’s shared knowledge base and alerts the subscribers to her data streams who may review and independently verify the results. If this particular materials system shows promise as a viable electrical insulator—and even if it does not—the data collected today will contribute to the growing online research data repositories from which her team developed their first model, a small act of solidarity in the modern scientific endeavor…*

**Table** **II** elaborates upon this hypothetical scenario in the context of the MITT framework. Achieving such visions will require persistent, well-coordinated efforts among a variety of stakeholders including materials researchers, software developers, original equipment manufacturers, and funding agencies. As a prerequisite, connecting systems-level expertise across disciplines requires shared language and communication tools. Domain-specific terminology and jargon tend to form lexical barriers that hinder cross-disciplinary collaboration and may obscure higher-level interdisciplinary commonalities. The classic materials tetrahedron paradigm presents a cogent visual depiction of the field of materials science, and revealing the underlying meta-framework enables the translation of this paradigm to information science.

**Table II:** To illustrate one of many possible applications of the MITT framework, aspects of the above narrative, specifically to the design of nanoparticle-polymer material systems for electrically insulating coatings, have been organized into the framework’s various dimensions.

**Dimensions of the meta-framework**					
*Activities*	*Arrangement*	*Qualities*	*Applicability*	*Validation*	*Viability*
**Materials tetrahedron**					
Processing	Structure	Properties	Performance	Characterization	Sustainability/criticality
Nanoparticle synthesis Particle surface modification Solution mixing Twin-screw extrusion Serial sectioning	Volume fraction Dispersion Interfacial area Graft density Entanglement Charge distribution	Dielectric breakdown strength Dielectric constant Dielectric loss Glass transition temperature Melting temperature Melt viscosity Charge mobility	Integration into encapsulation for high-voltage power transmission Reliability via enhanced charge trapping and self-healing mechanisms	X-ray diffraction (XRD), rheometry, thermogravimetric analysis (TGA), dynamic mechanical analysis (DMA), differential scanning calorimetry (DSC), broadband dielectric spectroscopy (BDS), electrostatic force microscopy (EFM), transmission electron microscopy (TEM), pulsed electro-acoustic (PEA) measurement	Designed recyclability and repurposing Assessed value of material selection within system design Life cycle and cost analysis Prognostics and prediction of performance lifetime Raw material traceability

**Information tetrahedron**					
Methods/workflows	Representations	Attributes	Efficacy	Evaluation	FAIR data principles
High-throughput experimentation Gradient libraries Image analysis Multi-scale simulations Bayesian optimization Data streams from APIs	Molecular data structures Binarized TEM images Voxelated reconstructions Stochastic models Processing, structure, characterization, and provenance metadata linked by ontology Interactive data visualization	Bias and uncertainty estimation Open-source software dependencies IoT-integrated hardware Data security and verifiability Intuitive user interface design	N-fold increase in research data output compared to traditional design space exploration Reduced time to integrate and scale up manufacturing	Validation of models through experiment Lab equipment integration Comparison against shared benchmarks Human–computer interaction assessment	Persistent identifiers Tiered access, data security Lab equipment, inventory, modeling DBs, analysis tools interoperate Reconfigurable, agile network of nodes Open-access, open-source integration

As a general approach, the meta-framework could apply to other systems-focused disciplines that couple with materials science to form closed feedback loops. For example, one can consider the coupling of materials science and bioengineering that occurs in bio-integrated materials.^87^ At a high-level, the systems-level meta-framework would apply to biomaterials engineering through consideration of activities (e.g., pathways), arrangement (e.g., geometry, organization), qualities (e.g., characteristics, traits), applicability (e.g., goodness of fit), validation (e.g., bioanalysis), and viability (e.g., longevity, compatibility) in terms of the biological system of interest. Instead of digital data and information flows, these interfaces might be mediated by sensing and modulation via biophysical or biochemical signals and actuation. However, we concentrate here on the convergence of materials and information science, leaving other combinatorial examples of joint frameworks open to future discussions.

With its high-level vantage point on materials and information science, the MITT framework provides a pedagogical launchpad and a groundwork for cross-disciplinary communication pertaining to infrastructure built around materials data. Faced with troves of data to potentially analyze, the challenge becomes how to organize researcher attention and computational resources around extraction of high-value information and the ultimate translation of this information into lasting solutions. The MITT framework extends the systems-oriented paradigm of the classic materials tetrahedron to encourage proficiency in tools that augment and accelerate materials discovery, design, development, and deployment through data science and informatics. By applying this framework to contextualize, translate, and direct various efforts in materials data and informatics, present and future generations of materials scientists and engineers will find themselves well-equipped to tackle the multifold challenges that arise in an increasingly complex, data-driven world.

## Footnotes

1 While Donahue replaces the materials tetrahedron with a square pyramid depiction, we have taken the artistic license to represent the aspect of Sustainability/Criticality as an encapsulating sphere to lend a “global” visual cue and preserve the recognizability of the classic materials tetrahedron.
